# Comparative analyses of cucumber (*Cucumis sativus* L.) cultivars with varying node formation rate in greenhouse

**DOI:** 10.1186/s12870-025-07587-3

**Published:** 2025-11-12

**Authors:** Eigo Ando, Kazuya Maeda, Naoto Aimi, Dong-Hyuk Ahn

**Affiliations:** 1 Institute of Vegetable and Floriculture Science, National Agriculture and Food Research Organization, 3-1-1 Kannondai, Tsukuba, Ibaraki, 305-8519 Japan; 2 Division of Vegetables, Miyazaki Prefectural Agricultural Experiment Station, 5805 Shimonaka, Sadowara-Cho, Miyazaki, 880-0212 Japan; 3 Current Affiliation: Graduate School of Science, Nagoya University, Furo-Cho, Chikusa, Nagoya, Aichi, 464-8602 Japan

**Keywords:** Cucumber, Dry-matter production, Dry-matter distribution, mRNA-seq, Shoot architecture

## Abstract

**Background:**

Shoot architecture is one of the fundamental traits in plants that diversifies their visual appearance. It is also an important agronomic trait as it substantially affects productivity and labor costs in crop species. Cucumber (*Cucumis sativus* L.) is a widely cultivated vegetable due to its economic value. As cucumber plants enter the vegetative and reproductive phases simultaneously, the shoot apical meristem continues to produce nodes bearing leaves, each capable of flower development in the axil. Therefore, the characteristics of node formation rate, which is generally recognized as a component of plant growth index ‘shoot vigor’ by growers, are expected to influence fruit yield. However, it remains unclear how and to what extent cultivar-dependent differences in node formation rate contribute to variation in yield.

**Results:**

We examined four Japanese cucumber cultivars (‘Josho-661’ [‘Josho’], ‘S-30’, ‘Green-Flush’, and ‘Yusho’), which are considered to differ in shoot vigor. ‘Josho’ exhibited the highest node formation rate and the highest fruit yield (36.3 kg m^–2^), whereas ‘Yusho’ developed fewer nodes and achieved the lowest yield (28.1 kg m^–2^). However, ‘S-30’ showed the highest dry-matter productivity, although ‘Josho’ exhibited better dry-matter partitioning to fruits. These results revealed that node formation rate and the consequent dry-matter allocation rather than dry-matter productivity can become limiting factors in cucumber yield. In addition, we conducted a comparative mRNA sequencing analysis of shoot tips to explore transcriptomic diversity and functional enrichment underlying the cultivars, which implied that variations in SAM maintenance gene, mitotic activity in SAM, and shoot tip temperature might be behind the difference in node formation rate.

**Conclusions:**

The growth analysis revealed that dry-matter productivity was not necessarily correlated with fruit yield and highlighted the potential impact of shoot vigor, particularly node formation rate, on cucumber fruit yield. Transcriptomic diversity observed in this study would genetically reinforce cucumber yield model.

**Supplementary Information:**

The online version contains supplementary material available at 10.1186/s12870-025-07587-3.

## Background

Plant species are often distinguished by their body architecture, which contributes to their diverse visual appearances. Shoot architecture arises from the organization and activity of specialized, undifferentiated cell clusters known as meristems, which generate new tissues and organs. The shoot apical meristem (SAM), located at the tip of the shoot, drives vertical growth by producing a structural unit called the phytomer, which includes a node bearing a leaf, an axillary meristem, and an internode. The axillary meristem, typically formed in each leaf axil, promotes lateral growth by generating branches. During the transition from the vegetative to the reproductive phase, the SAM becomes the inflorescence meristem, producing flowers or flower-bearing shoots [1].

Shoot architecture also plays a critical role in agriculture, as it influences crop productivity and management efficiency. Consequently, several architectural traits have been selected during the domestication and improvement of crop species. Cucumber (*Cucumis sativus* L.) is one of the most economically important fruit-bearing vegetable crops [2]. Its wild ancestor, *C. sativus* var. *hardwickii*, exhibits increased branching, a weak stem, small leaves, and reduced fruit size compared to domesticated cultivars [3], suggesting that these traits have been subject to artificial selection. For example, increased planting density during domestication may have enhanced the shade avoidance response, thereby suppressing axillary bud outgrowth. This response is associated with the acquisition of light response elements in the promoter region of *BRANCHED1*, a central negative regulator of branching, in domesticated cultivars [4]. Reduced branching decreases labor costs associated with pruning and enhances the allocation of resources, including nutrients, carbon, and light, for photosynthesis and yield. Understanding of shoot architecture is therefore essential for breeding high-yield, low-labor-input cultivars.

Node formation is thought to be influenced by the activity and identity regulation of the SAM. Several key genes function at the shoot tips in cucumber plants. TERMINAL FLOWER1 (CsTFL1) and CsTFL1d, members of the phosphatidylethanolamine-binding protein (PEBP) family [5–8], contribute to indeterminate growth by interacting with NEGATIVE ON TATA-less 2a (CsNOT2a), a global negative transcriptional regulator [9], and the bZIP transcription factor FD PARALOG (CsFDP), thereby suppressing floral meristem induction [10, 11]. In contrast, FLOWERING LOCUS T (CsFT), another PEBP family protein, promotes determinate growth by interacting with CsFD through G-box factor 14–3-3 proteins [10]. The transcription factor LEAFY (CsLFY) acts in concert with the homeodomain protein WUSCHEL (CsWUS) and likely with the F-box protein UNUSUAL FLORAL ORGANS (CsUFO) to maintain stem cell identity and regulate floral meristem differentiation [12]. Additionally, the GATA3 transcription factor HANABA TARANU (CsHAN1/2) has been shown to mediate the expression of CsWUS and the knotted-like homeobox genes SHOOT MERISTEMLESS (CsSTM) and BREVIPEDICELLUS (CsBP), thereby contributing to SAM maintenance [13].

Unlike most flowering plant species, cucumber plants produce leaf-bearing nodes from the SAM while simultaneously generating flowers from the axils of these nodes during much of their life cycle [3]. Therefore, shoot architecture, particularly the node formation rate (NFR) and total number of nodes, may influence fruit number per shoot and the consequent fruit yield. This idea is theoretically supported by crop yield model for fruit-bearing vegetables [14, 15]. In general, the yield model provides a theoretical framework for understanding how yields are determined throughout a plant’s lifetime as follows. Fresh fruit yield is determined according to dry fruit yield and the dry-matter (DM) content of the fruit. In turn, dry fruit yield depends on total DM production (TDM) and the proportion of DM allocated to the fruit. Then the sink strength of each fruit and the number of fruits per shoot, which should be influenced by the total node count and NFR, determine the amount of DM partitioned to the fruit [14]. On the other hand, TDM is governed by the total amount of light intercepted by the canopy and the light use efficiency (LUE), both of which are influenced by individual leaf photosynthetic capacity, light-absorption traits of the canopy (such as the light extinction coefficient), and the leaf area index (LAI) [14]. Previous studies underscore the importance of DM productivity and its allocation for fruit yield in vegetable crops. A comparison of Dutch and Japanese tomato cultivars demonstrated that increased LUE has contributed to yield improvement in the Netherlands over the past 50 years, mainly due to a reduced light extinction coefficient and enhanced leaf photosynthetic rate [16]. In F1 hybrid cucumbers, yield advantages inherited from parental lines were attributed to enhanced vegetative growth, DM accumulation, and fruit setting [17]. We previously evaluated nine cucumber cultivars that differed in TDM and DM allocation to fruit; these traits were associated with differences in LUE and fruit number per shoot, respectively [15]. However, the direct contribution of NFR to yield remains unclear in terms of individual yield components.

Although cucumber cultivars exhibit considerable diversity, the extent to which variation in shoot architecture influences fruit yield remains insufficiently explored. NFR is generally recognized as a component of robustness and rapidness of shoot growth, which is utilized as the plant growth index so-called shoot vigor, by growers. In this study, we examined four Japanese cucumber cultivars considered to exhibit differences in shoot vigor. We assessed stem growth and confirmed that these cultivars exhibit distinct NFRs. Yield differences were consistent with the total number of nodes formed during the experiment. Then, we compared DM production and its partitioning among the cultivars. Notably, DM productivity was not always correlated with yield, and variation in the NFR emerged as a primary factor contributing to yield differences. In addition, we performed transcriptomic analysis using messenger RNA sequencing (mRNA-seq) of shoot tips to explore the genetic diversity underlying the cultivars. We compared the expression levels of previously characterized meristem identity-regulating genes and conducted functional enrichment analysis of differentially expressed genes (DEGs). The findings of this study highlight the potential impact of shoot vigor, specifically NFR, on fruit yield in cucumber plants and would provide a genetic framework that supports the cucumber yield model.

## Methods

### Plant materials and growth conditions

Four cucumber genoecious cultivars, ‘Josho-661’ (hereinafter, ‘Josho’), ‘S-30’, ‘Yusho’ (Saitama Genshu Ikuseikai Co., Ltd., Japan), and ‘Green-Flush’ (hereinafter, ‘G-Flush’; Tokiwa Co., Ltd., Japan) were investigated in this study. Seedlings grafted onto the rootstock pumpkin cultivar ‘RK-3’ (for ‘Josho’, ‘S-30’, and ‘Yusho’; Saitama Genshu Ikuseikai Co., Ltd.) or ‘Zokkon’ (for ‘G-Flush’; Tokiwa Co., Ltd.) were commercially obtained from JA AGRISEED (Miyazaki, Japan). The seedlings were transplanted into rockwool (Cultilene, Rijen, Netherlands) placed in a glass greenhouse (width × depth × height = 6.0 m × 20.0 m × 2.0 m) at the Miyazaki Prefectural Agricultural Experiment Station, Japan, on October 15, 2021. The row and plant spacing were 1.5 m and 0.3 m, respectively. Plants were grown as single shoots for 17 days, then allowed to branch and grow as double shoots, resulting in a planting density of 4.44 stems m^–2^. Then, lateral branches were removed throughout the experiment. They were cultivated for 228 days following transplanting, with irrigation using a mixture of TF Full-mix A and TF Mix B nutrient solutions (Toyotane, Toyohashi, Japan). Greenhouse conditions were regulated by the Greenhouse Horticulture SaaS system (Fujitsu, Kawasaki, Japan), with heating pipe and ventilation settings maintained at 15 °C and 28 °C, respectively. Relative humidity in the greenhouse was maintained at approximately 75%. Total global solar radiation for Miyazaki was obtained from the Japan Meteorological Agency. (https://www.jma.go.jp/jma/indexe.html). The greenhouse light transmission rate was 0.51 MJ MJ^–1^, measured using a quantum sensor (LI-190R; Li-Cor, Lincoln, NE, USA) before the start of the experiment.

### Cucumber harvesting

Cucumbers were harvested daily from 39 days after transplanting (DAT) until the end of the experiment. Fresh yield and fruit number were estimated using nine plants per cultivar. Aborted fruits, defined as those with a fresh weight below 3.0 g fruit^–1^, were excluded from the yield data. The ratio of aborted fruits and the female flowering rate were calculated based on the total fruit number, including aborted fruits. The female flowering rate was determined by dividing the total fruit number by the final node number, excluding premature fruits due to their limited occurrence. Fruit dry yield was estimated by multiplying fresh yield by fruit DM content, which was determined from a separate cultivation conducted by the National Agriculture and Food Research Organization (NARO) in 2024 (see Supplemental Methods).

### Growth analyses

Node numbers and the positions of open female flowers were recorded from 38 to 227 DAT. The increase in node number was calculated by subtracting the initial count on 38 DAT from each subsequent observation. This value was expressed as a function of accumulated daily average temperature starting from 38 DAT. Six plants per cultivar were used in the analysis.

### Destructive measurements

The entire aboveground portion of the cucumber plants (leaves, stems, and fruits) was collected at 0, 45, 66, 105, 142, and 228 DAT, and fresh weights were recorded. Then, the tissues were dried at 70 °C for at least 72 h to determine dry weights. Aboveground TDM was calculated by adding the dry weights of harvested fruits and trimmed leaves to the measured dry biomass. DM production between two time points was estimated by subtracting the mean dry weight at the earlier timepoint from the dry weight of individual plants at the subsequent timepoint. Three plants were sampled at each timepoint, except for the final measurement, in which nine plants were used.

### Estimation of LAI, daily light interception, and LUE

LAI was non-destructively estimated by calculating the product of leaf width and length, as follows:

1 $$\text{Individual leaf area}=0.8072\times \text{Leaf width}\times \text{Leaf length}-1.6083$$

Non-destructive measurements were conducted weekly from 32 to 228 DAT. Three plants per cultivar were used for these measurements. Daily LAI between measurements was estimated via interpolation. LAI during 0–32 DAT was estimated by extrapolation, assuming LAI at transplanting to be zero. IL was calculated as follows:

2 $${IL}_{n}={SR}_{n}\times T\times Ratio of PAR\times \left(1-{e}^{-k\times {LAI}_{n}}\right)$$

where *T* is the greenhouse transmission rate. The ratio of photosynthetically active radiation (PAR) to solar radiation was assumed to be 0.5 MJ MJ^–1^ [18]. The light extinction coefficient (*k*) of the cucumber canopy was estimated to be 0.9 based on a preliminary experiment using ‘Josho’ and ‘S-30’ according to a previously described method [19]. LUE was estimated by regression analyses between the cumulative amount of intercepted light and TDM minus the initial dry weight (Fig. S1) as described previously [16].

### RNA extraction and mRNA-seq

Plants cultivated as described in the Supplemental Methods were used for this experiment. Shoot tips (up to approximately 100 mg) were harvested on 108 DAT, immediately frozen in liquid nitrogen, and stored at –80°C until RNA extraction. Four biological replicates were used for the analysis. Total RNA was extracted using the RNeasy Plant Mini Kit (Qiagen, Hilden, Germany), and contaminating DNA was removed using the RNase-Free DNase Set (Qiagen), following the manufacturer’s instructions. mRNA was purified using a poly(A)-selection method for Illumina, and cDNA libraries were prepared with the NEBNext Ultra II Directional RNA Library Prep Kit for Illumina (Illumina, San Diego, CA, USA). Sequencing was performed on the NovaSeq 6000 system (Illumina). After filtering out low-quality reads, short-read alignment was conducted against the *Cucumis sativus* L. cv. ‘Chinese long’ reference genome v3, available from the cucurbit genomics database CuGenDB v2 (http://cucurbitgenomics.org/v2/ [20]) using Hisat2 [21] with the default parameters.

### Bioinformatics

RNA-seq data were analyzed using R software [22]. DEGs between ‘Josho’ and the other cultivars were identified using the *edgeR* v4.2.1 package [23]. Genes with low read counts were filtered using the ‘filterByExpr’ function with the default parameters, resulting in 19,064 genes retained for analysis. A quasi-likelihood F test was performed, and genes with |log_2_(fold change)|≥ 1.0 and FDR < 0.05 (‘Josho’ vs. the others) were considered DEGs. Common DEGs among ‘S-30’, ‘G-Flush’, and ‘Yusho’ were subjected to GO enrichment analysis using the *clusterProfiler* package [24]. A cucumber-specific OrgDb package was constructed from the gene annotation file available in the database using the *AnnotationForge* package [25]. GO terms with FDR < 0.05 were considered significantly enriched. Accession numbers of meristem identity-regulating genes reported in previous studies were re-identified in the current reference genome by BLAST searches based on their amino acid sequences (Table S1). Heatmaps were generated using the ‘heatmap.2’ or ‘corrplot’ functions in the *gplots* and *corrplot* packages, respectively [26, 27]. Venn diagrams were created using the ‘VennDiagram’ package [28].

### Statistical analyses

Statistical analyses were conducted using R software. Differences among means were assessed using Tukey’s test with the *multcomp* package [29]. Regression coefficients were compared using the *emmeans* package [30]. Correlations among yield components were analyzed using the *psych* package with Benjamini–Hochberg adjustment [31] and visualized using the *corrplot* package. Significance was evaluated at a level of *P* < 0.05.

## Results

### Cucumber cultivars characterized by NFR

**Fig. 1:**
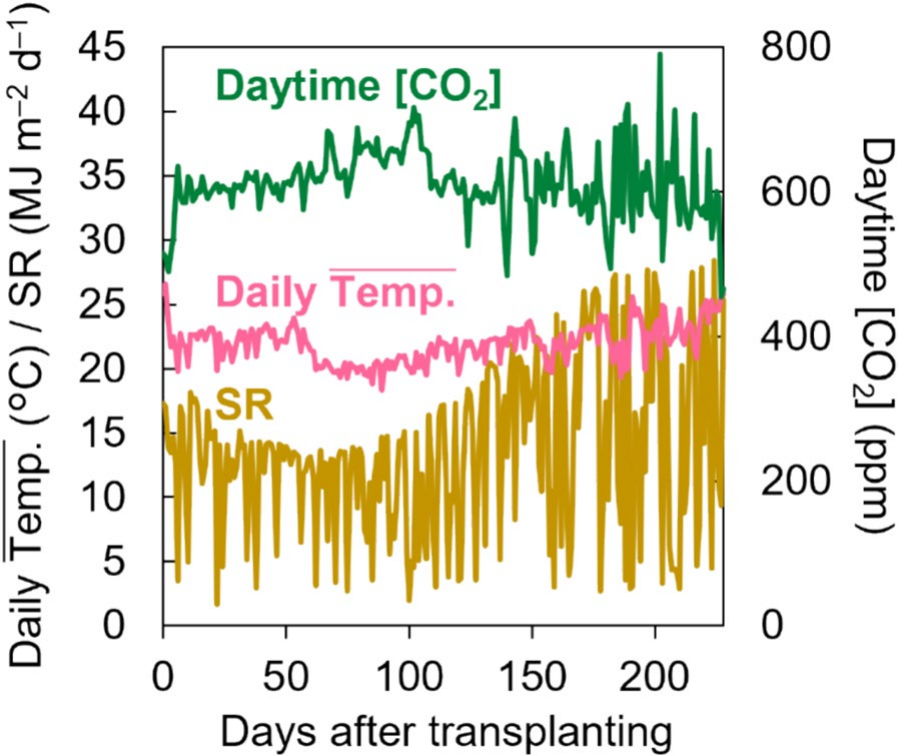
Growth conditions during cultivation. Daytime CO_2_ concentration, daily average temperature within the greenhouse, and cumulative daily solar radiation are shown

**Fig. 2:**
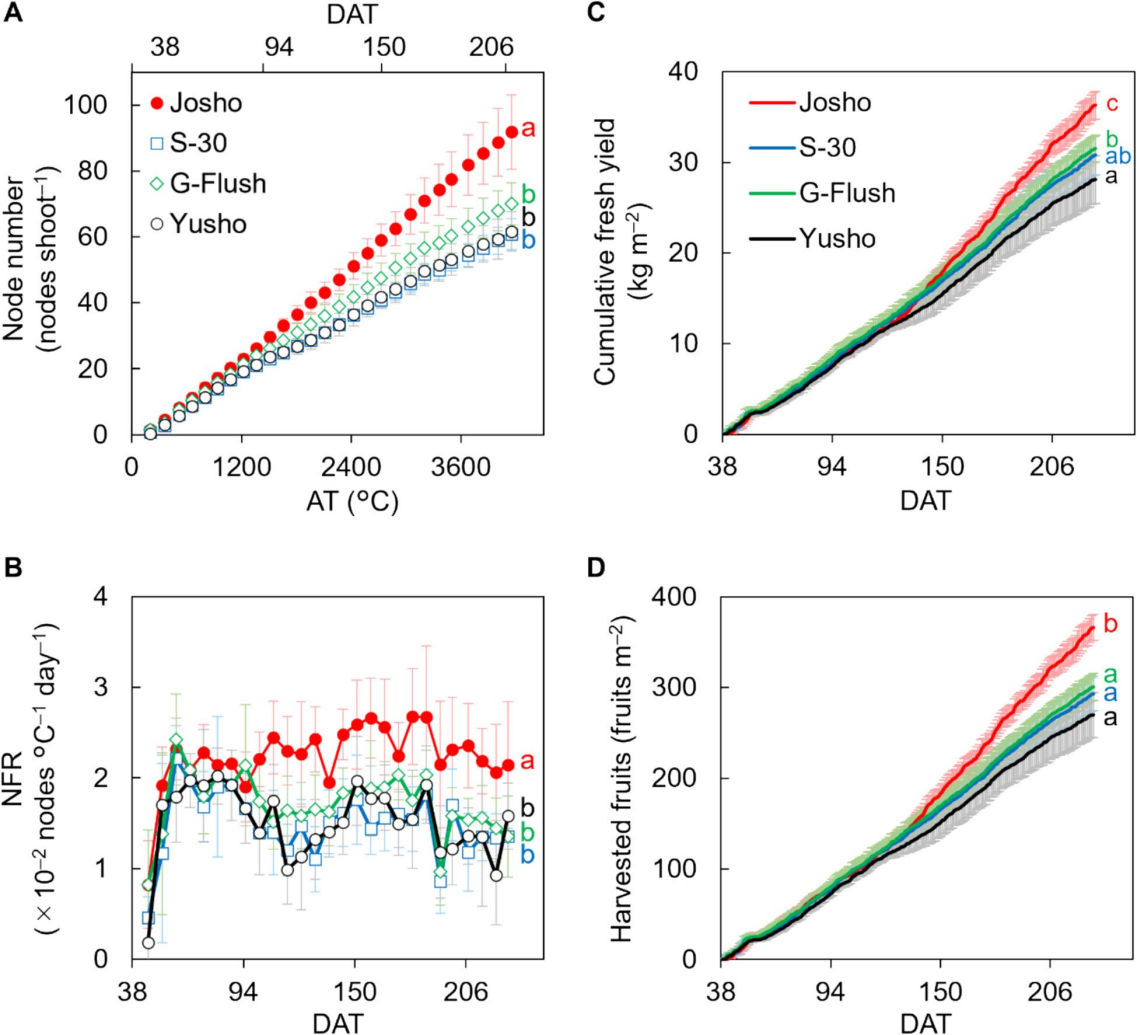
Node formation rate (NFR) and fruit yield of each cultivar. **A** Changes in the number of nodes. Node number was expressed as a function of accumulated temperature (AT). **B** Changes in the NFR in response to AT. **C** Cumulative fresh yields. **D** Total amount of harvested fruit. Data are means of six (**A**, **B**) or nine (**C**, **D**) plants with 95% confidence intervals (CIs). Different letters indicate significant differences in the final value (**A**, **C**, and **D**) or average NFR throughout the experiment (**B**) (*P* < 0.05, Tukey’s test)

To demonstrate the potential effects of cultivar-dependent variation in shoot architecture on cucumber yield, we selected four commercially available Japanese cultivars (‘Josho’, ‘S-30’, ‘G-Flush’, and ‘Yusho’), which are recognized for their different levels of vigor. The experiment was conducted in 2021, with plants grown in a greenhouse under the conditions shown in Fig. 1. We assessed node numbers and found that ‘Josho’ had formed more nodes than all other cultivars by the end of cultivation period (Fig. 2A). Then, we analyzed changes in the NFR, calculated as the number of nodes formed per unit of daily average temperature, throughout the experiment (Fig. 2B). The NFR was higher in ‘Josho’ than in the other cultivars, particularly ‘S-30’ and ‘Yusho’. The mean NFRs were 2.2 × 10^–2^ nodes °C^–1^ day^–1^ for ‘Josho’ and approximately 1.5 × 10^–2^ nodes °C^–1^ day^–1^ for the other cultivars. A similar trend was observed in a separate short-term experiment conducted in a different greenhouse and year (Fig. S2), suggesting that the observed differences are cultivar-dependent rather than condition-dependent. We also noted cultivar-specific variation in the position of open female flowers along the stem. In ‘Josho’, open female flowers tended to appear at nodes farther from the shoot tip compared to the other cultivars (Fig. S3), implying more rapid stem growth. These findings indicate that the cultivars are distinguishable by their shoot architecture, particularly in terms of NFR and node number.

### Comparison of fruit yield among cultivars

Final fresh yield was highest in ‘Josho’ (approximately 36.3 kg m^–2^), consistent with its elevated NFR compared to the other cultivars (Fig. 2C). The number of harvested fruits followed a similar pattern: ‘Josho’ produced around 360 fruits per unit area, whereas the other cultivars yielded approximately 300 or fewer (Fig. 2D). Mean harvested fruit weight was around 100 g fruit^–1^ (Fig. S4A). The ratio of aborted fruits, which were excluded from yield calculations, was higher in ‘Josho’ (approximately 7.4%) than in the others (1.0% or less, Fig. S4B). Female flowering rates were broadly similar across cultivars, although ‘G-Flush’ exhibited a slightly lower rate (Fig. S4C). These findings suggest that yield differences were primarily driven by variation in node formation, rather than by fruit abortion or female flowering frequency in the cultivars examined.

### DM distribution

**Fig. 3:**
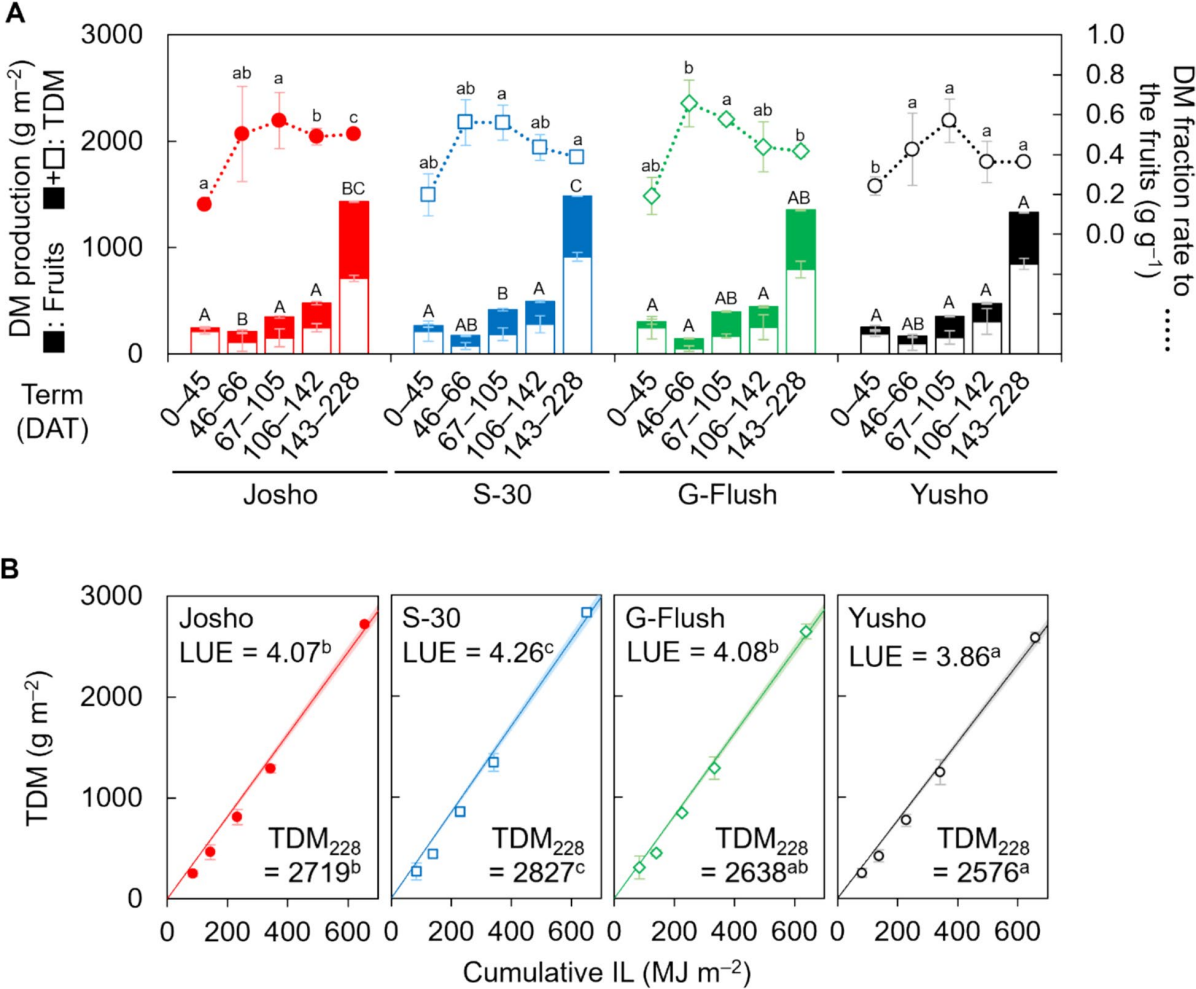
Dry-matter (DM) partitioning and productivity of each cultivar. **A** DM production and distribution of fruits analyzed for five intervals between destructive measurements. **B** Light use efficiency (LUE). Lines and shading indicate regression lines for TDM and their 95% CIs, respectively. LUE was estimated as a regression coefficient. Total DM (TDM) at the end of cultivation (TDM_228_) is also shown. Data are means of three (first to fourth destructive measurements) or nine (last measurement) plants with 95% CIs. Different letters indicate significant differences (*P* < 0.05; Tukey’s test) within the same intervals (A, lower case: DM fraction rate; upper case: TDM) or among cultivars (**B**)

Next, we compared the cultivars in terms of how DM was partitioned to fruit. The experimental period was divided into five terms based on the timing of destructive measurements (term 1: 0–45 DAT; term 2: 45–66 DAT; term 3: 66–105 DAT; term 4: 105–142 DAT; term 5: 142–228 DAT), and DM partitioning was analyzed for each term. As shown in Fig. 3A, DM partitioning in ‘Josho’ during the first term was 0.15 g g^–1^ and remained around 0.5 g g^–1^ in later terms. In contrast, the other cultivars, particularly ‘Yusho’, showed higher early DM partitioning (approximately 0.2 g g^–1^) but failed to maintain these levels beyond the third term. In the final term, DM partitioning in ‘S-30’, ‘G-Flush’, and ‘Yusho’ declined to approximately 0.4 g g^–1^. As a result, the cumulative fruit dry yield relative to TDM in ‘Josho’ continued to increase, reaching 0.48 g g^–1^ by the end of the experiment. In comparison, the other cultivars peaked at around 0.44 g g^–1^ on 105 DAT and declined thereafter (Fig. S5A). Additionally, DM content per fruit was comparable across cultivars, confirming that fruit quality was consistent (Fig. S5B). Together, these results indicate that reduced node formation suppressed DM partitioning to fruit.

### DM productivity

**Fig. 4:**
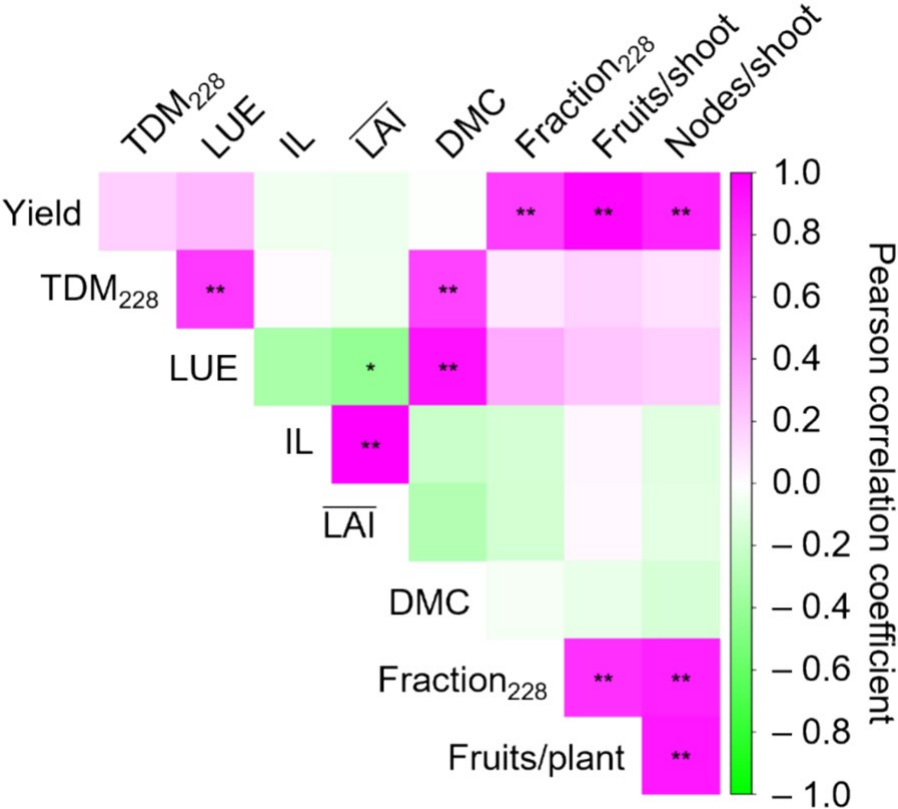
Assessment of the four cucumber cultivars based on yield components. Data obtained for each cultivar were pooled and correlations among yield components were analyzed. The correlation matrix is visualized as a heatmap. Asterisks indicate significant correlations (*P < 0.05, **P < 0.01; Benjamini–Hochberg adjustment). DMC, DM content of fruits

Although yield differences among the cultivars could be attributable to variation in both shoot architecture and DM productivity, this did not appear to be the case in the present experiment. TDM across the five experimental terms was largely comparable among cultivars, except during the third and final terms (Fig. 3A). Notably, ‘S-30’, but not ‘Josho’, tended to exhibit higher TDM. In the third and final terms, TDM in ‘S-30’ reached 417 and 1,484 g m^–2^, respectively, while the values for ‘Josho’ were 348 and 1,431 g m^–2^. ‘Yusho’ showed the lowest TDM in the final term (1,329 g m^–2^). As LAI was maintained at approximately 2.4 m^2^ m^–2^ across all cultivars, cumulative IL was estimated to be similar among cultivars (around 650 MJ m^–2^; Fig. S6). Consequently, LUE varied as shown in Figure S7, with ‘S-30’ exhibiting the highest LUE (4.26 g MJ^–1^), followed by ‘G-Flush’ (4.08 g MJ^–1^), ‘Josho’ (4.07 g MJ^–1^), and ‘Yusho’ (3.86 g MJ^–1^). Similar trends were observed in TDM at the end of the experiment (Fig. 3B). The discrepancies between yield and TDM or LUE suggest that DM productivity had only a marginal effect on yield differences among cultivars. Indeed, correlation analysis of the yield components revealed no significant relationship between yield and DM productivity. In contrast, a strong correlation was observed between node formation and fruit yield (Fig. 4). These findings demonstrate that cultivar-dependent shoot architecture can influence fruit yield independently of DM productivity in cucumber plants.

### Transcriptomic diversity among cultivars

**Fig. 5:**
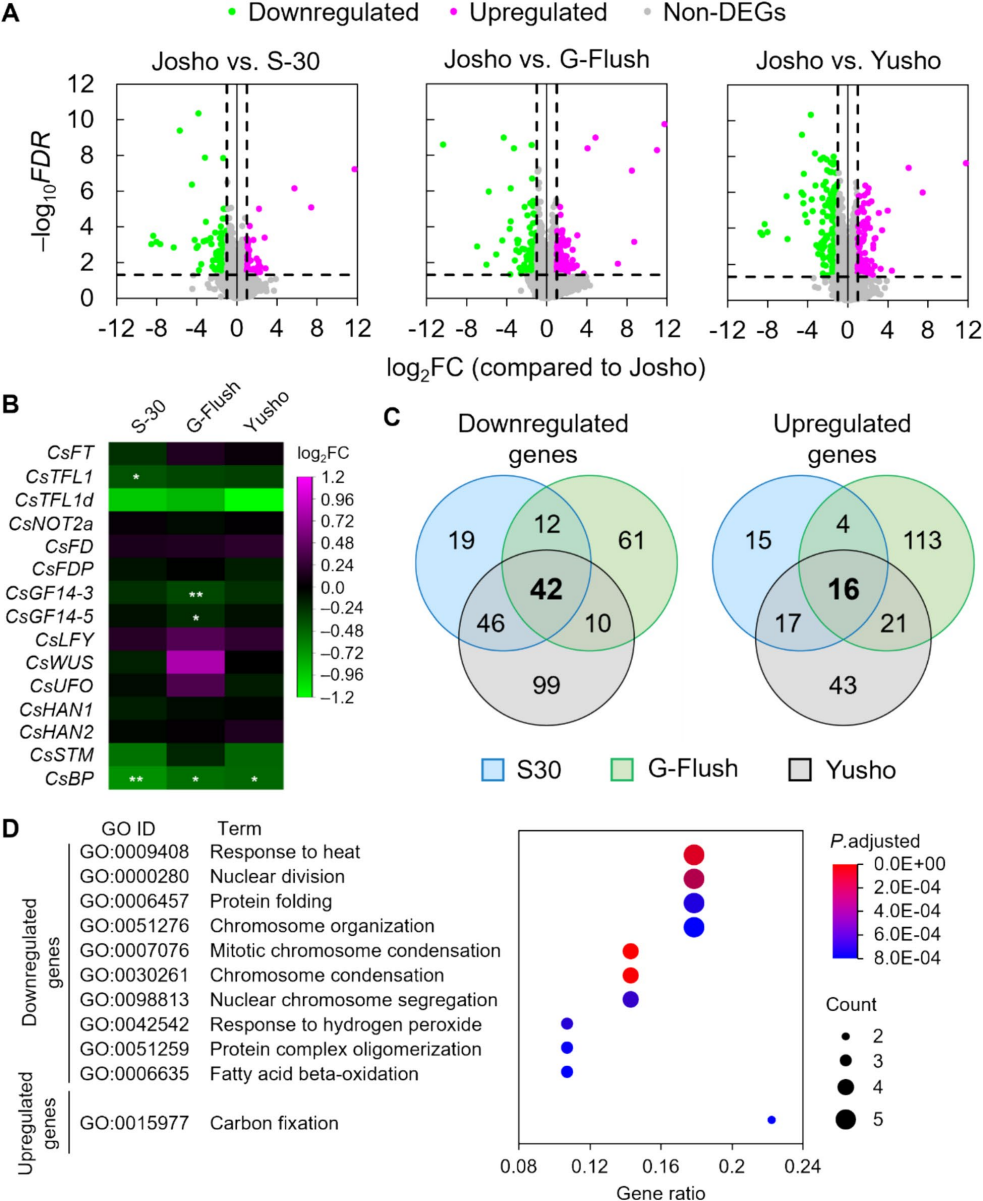
Comparison of the transcriptome in shoot tips between the ‘Josho’ cultivar and other cultivars via mRNA sequencing. (**A**) Volcano plots representing each pairwise comparison. Horizontal and vertical axes represent fold changes (FCs) and false discovery rates (FDRs), respectively, in logarithmic scale. See also Figure S8. (**B**) FCs of genes involved in shoot apical meristem (SAM) maintenance and floral meristem differentiation reported in previous studies (Table S1). Asterisks indicate the FDR and FC of each gene (*FDR < 0.05; **FDR < 0.01). (**C**) Venn diagrams representing the numbers of genes down- or upregulated in the ‘S-30’, ‘G-Flush’, and ‘Yusho’ cultivars compared to ‘Josho’. Common differentially expressed genes (DEGs) are shown in bold. (**D**) Gene Ontology (GO) enrichment analysis of biological processes. The top 10 GO terms are shown for the common down- and upregulated genes in ascending order of *P* values adjusted using the Benjamini–Hochberg method. Normalized count data and overall GO enrichment analysis results are provided in Datasets S1–S4

In cucumber plants, nodes are produced by the SAM while flowers develop from the leaf axil at each node [3], underscoring the importance of SAM activity for yield. Certain meristem identity-regulating genes determine whether the SAM exhibits determinate or indeterminate growth in *Cucumis sativus* [3]. These findings suggest genetic diversity among the shoot tips of the four cultivars. To explore this possibility, we performed mRNA-seq using RNA extracted from their shoot tips. We identified more than 500 differentially expressed genes (DEGs), assessed using the criteria |fold change|> 2 and false discovery rate (FDR) < 0.05, in pairwise comparisons between ‘Josho’ and the other cultivars (Figs. 5 and S8, and Dataset S1). We also examined the expression of genes previously reported to regulate SAM identity (Table S1), including PEBPs involved in floral meristem induction (CsFT, *CsTFL1*, *CsTFL1d*); subunits of protein complexes including *CsFT* or *CsTFL1/CsTFL1d* (*CsNOT2a*, *CsFD*, *CsFDP*, *CsGF14-3*, *CsGF14-5*); and transcription factors and their interactors involved in SAM maintenance and floral meristem differentiation (*CsLFY*, *CsWUS*, *CsUFO*, *CsHAN1*, *CsHAN2*, *CsSTM*, *CsBP*). We initially expected some of these genes to meet the criteria for differential expression, but none qualified as DEGs. However, *CsTFL1* in ‘S-30’, *CsGF14-3* and *CsGF14-5* in ‘G-Flush’, and *CsBP* in all three cultivars showed modest reductions (approximately 30% on average) compared to ‘Josho’ (Dataset S2). To further investigate physiological differences, we conducted Gene Ontology (GO) enrichment analysis of DEGs common among ‘S-30’, ‘G-Flush’, and ‘Yusho’. We selected 42 downregulated and 16 upregulated genes and analyzed their enrichment in biological processes. Only one GO term was significantly enriched among upregulated genes, related to carbon fixation. This term included two DEGs: *CsaV3_4G001210*, which encodes a putative CP12 domain-containing protein, and *CsaV3_6G048750*, a putative *RbcX* homolog (Fig. S9A and Datasets S3 and S4). In contrast, downregulated DEGs were enriched for GO terms associated with heat response, cell division, and β-oxidation. These included putative condensin proteins (*CsaV3_UNG044290*, *CsaV3_UNG075600*, *CsaV3_UNG088700*, *CsaV3_UNG225770*), heat-shock proteins and co-chaperones (*CsaV3_1G035820*, *CsaV3_1G035830*, *CsaV3_1G044020*, *CsaV3_3G006720*, *CsaV3_5G026520*), a multiprotein bridging factor 1c (MBF1c) homolog (*CsaV3_2G002310*), an ankyrin repeat-containing protein (*CsaV3_1G010260*), and 3-hydroxyacyl-CoA dehydrogenase proteins (*CsaV3_6G023960*, *CsaV3_6G024070*, *CsaV3_024110*) (Fig. S9B and Datasets S3 and S4). These findings indicate that the shoot tips of the four cultivars differ at the transcriptional level, which may reflect genetic factors underlying the observed variation in NFR.

## Discussion

### Potential effects of the NFR on fruit yield

Because cucumber flowers develop at each leaf axil on the corresponding node, shoot architecture, particularly node formation, is expected to influence fruit yield; however, theoretical explanations and supporting evidence have been limited. In this study, ‘Josho’ exhibited the highest yield, accompanied by the highest NFR among the cultivars (Fig. 2). Notably, differences in NFR began to emerge around 95 DAT, followed by a widening yield gap from approximately 120 DAT. Despite its high yield, mean harvested fruit weight in ‘Josho’ was slightly smaller than those of the others (Fig. S4A). This cultivar also showed a higher rate of fruit abortion (Fig. S4B). Although fruit set can be influenced by male and female flower differentiation [32], we observed no substantial differences in female flowering rates among the cultivars (Fig. S4C). These findings suggest that variation in fruit set among the tested cultivars was primarily driven by cultivar-dependent NFR, rather than by fruit weight, abortion, or female flowering. As in other fruit-bearing species such as tomato, total DM production and its partitioning to fruit determine fruit yield in cucumber [5, 8]. We compared DM productivity across cultivars and found that ‘Josho’ did not necessarily exhibit high LUE (Fig. 3 and Fig. S7). Instead, ‘Josho’ was distinguished by its ability to sustain a high ratio of DM partitioning to fruit. Final yield was strongly correlated with the DM fraction allocated to fruit, which in turn was associated with fruit number and node number per shoot (Fig. 4). These results indicate that cultivar-level variation in NFR may influence yield by altering fruit number along the shoot and the resulting DM partitioning.

Fruit yield is influenced by both source–sink balance and fruit set [7]. In this study, we observed that ‘S-30’ exhibited high DM productivity despite its low NFR and comparable LAI (Figs. 2 and S6), suggesting that source strength was higher in ‘S-30’ than in the other cultivars (Fig. 3). In contrast, the numbers of harvested fruits in ‘S-30’, ‘G-Flush’, and ‘Yusho’ were 18–26% lower than in ‘Josho’ (Fig. 2D), while the corresponding reductions in total DM fraction were more modest, at 10–20% (Fig. S5A). Given that the DM fraction is determined by both the number of fruits per shoot and the sink strength of individual fruits [5], these results imply that fruit sink strength in the three cultivars was higher than in ‘Josho’. Together, these differences in source and sink strength may have led to cultivar-specific source–sink balances, which were potentially suboptimal in ‘S-30’, ‘G-Flush’, and ‘Yusho’ for achieving high yield. Further analysis of individual fruit growth is necessary to clarify this relationship. In addition, we cannot exclude a possibility that compatibility issue between the shoot and rootstock used in the growth analysis may influence the DM allocation and source-sink balance in entire plants, although the rootstock cultivars were recommended by the breeders.

### Putative genetic background for cultivar-dependent variation in NFR

We hypothesize that genetic diversity underlies the variation in NFR among the cultivars. Although none of the previously characterized genes related to SAM regulation were identified as DEGs in our mRNA-seq analysis, several genes exhibited marginal decreases in ‘S-30’, ‘G-Flush’, and/or ‘Yusho’ compared to ‘Josho’ (Fig. 5). In the model species *Arabidopsis thaliana*, *AtBP* has been shown to function redundantly with *AtSTM* in meristem maintenance [33–35]. A comparison of the spatial expression patterns of meristem genes, including *CsBP*, in wild-type and *CsHAN1*-RNAi cucumber lines, suggested that SAM development in *Cucumis sativus* is mediated by the expression of *CsBP*, *CsSTM*, and *CsWUS* [13]. The same study suggested that suppression of *CsHAN1* resulted in the decrease in growth rate and flower buds in cucumber [13], from which decrease in NFR is inferred. Thus, it is noteworthy that *CsBP* expression was downregulated in ‘S-30’, ‘G-Flush’, and ‘Yusho’, and further genetic and biological validation is needed to clarify its potential contribution to the yield gap among cultivars.

GO enrichment analysis revealed physiological differences in shoot tips among the cultivars. Downregulation of genes associated with cell division suggests that mitotic activity in ‘S-30’, ‘G-Flush’, and ‘Yusho’ is lower than in ‘Josho’. In maize, SAM volume is correlated with agronomic traits such as leaf node count [36], implying that reduced NFR in the three cultivars may result from diminished mitotic activity and a consequent decrease in SAM volume. Additionally, downregulation of heat response-related genes indicates that shoot tip heat sensitivity and/or its temperature vary among cultivars. A previous study suggested that shoot tip temperature, rather than temperature in other organs, positively correlates the leaf initiation rate in cucumber [37], and that high transpiration rates in the shoot tip contribute to lower tissue temperatures [38]. It could be considered that shoot tips of ‘Josho’ is more heat tolerant to exhibit higher NFR. Alternatively, cultivars other than ‘Josho’ might exhibit low shoot tip temperature that suppressed their NFR. Comparative analyses of shoot tip temperature, transpiration capacity, and microscopic SAM morphology among cultivars would be valuable to further elucidate the physiological basis of NFR variation.

## Conclusion

**Fig. 6:**
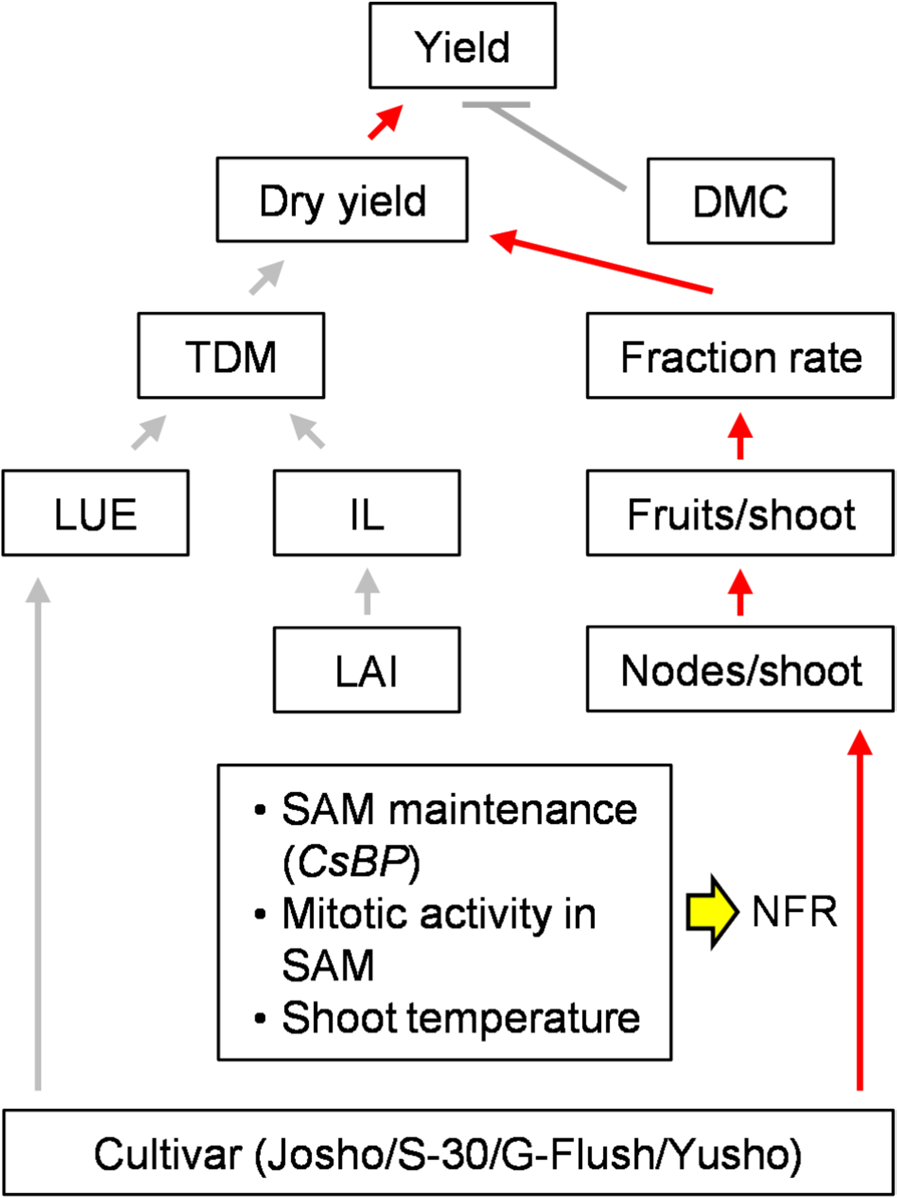
Hierarchy of cucumber yield components. Red lines indicate primary processes underlying yield differences among the four cultivars. Arrows and T-bars represent positive and negative effects, respectively. Yellow arrow denotes a putative effects of cultivar-dependent factors on node formation rate (NFR)

Cucumber cultivars with different NFRs were primarily distinguished by their ratio of DM partitioning to fruit, rather than by overall DM productivity. This variation was accompanied by transcriptional differences related to SAM maintenance, heat shock response, and cell division, which would be a potential genetic explanation supporting a cucumber yield model structured around a hierarchical framework of yield components (Fig. 6).

## Supplementary Information

Supplementary Material 1. 

Supplementary Material 2. 

Supplementary Material 3. 

## Data Availability

mRNA-seq data was deposited in the DNA Data Bank of Japan (DDBJ) with an accession number PRJDB35807. The other data underlying this article are available upon reasonable request to the corresponding author.

## Abbreviations

SAM: Shoot apical meristem
DM: Dry-matter
TDM: Total dry-matter production
LUE: Light use efficiency
LAI: Leaf area index
NFR: Node formation rate
mRNA-seq: Messenger RNA sequencing
DEG: Differentially expressed genes
DAT: Days after transplanting
GO: Gene ontology
