# Supplementary material for: Comparative analyses of cucumber (Cucumis sativus L.) cultivars with varying node formation rate in greenhouse
Source: BMC Plant Biol. 2025 Nov 12;25:1547. doi: 10.1186/s12870-025-07587-3 (PMC12613712; doi:10.1186/s12870-025-07587-3)
Supplement: Supplementary file 1 — Supplementary Material 1. [file 12870_2025_7587_MOESM1_ESM.pdf]

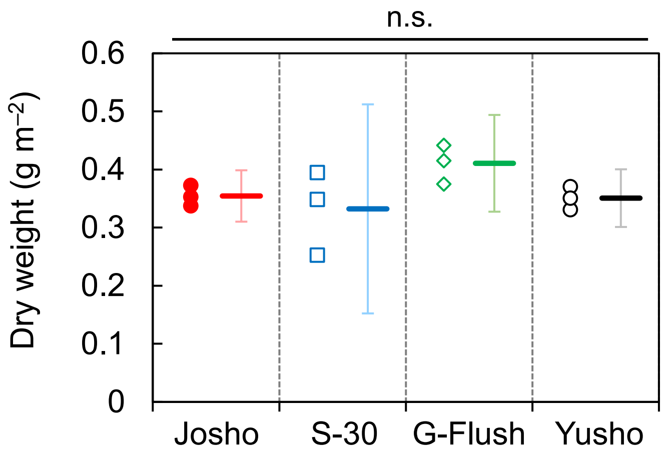

Figure S1. Dry weight of cucumber plants at transplanting. Each plot represents an individual plant (n = 3 per cultivar). Horizontal bars and error bars represent means and 95% CIs, respectively. Initial dry weights were not significantly different among cultivars (n.s., $P > 0.05$; Tukey's test).

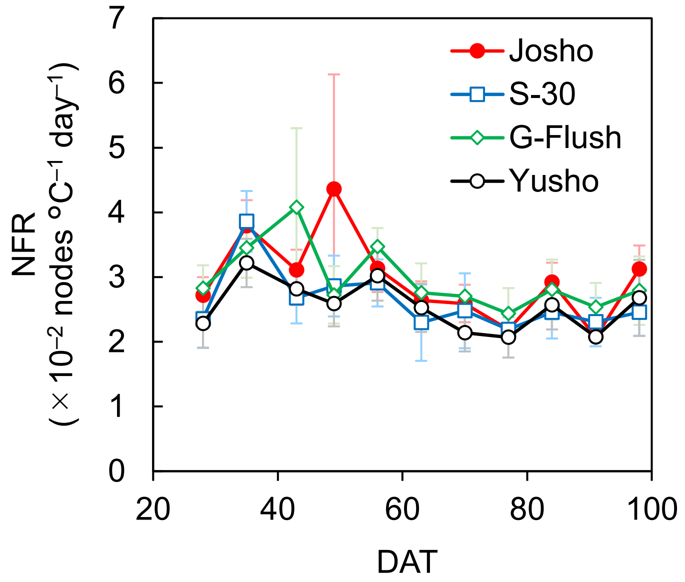

Figure S2. Changes in the NFR in an additional experiment. The NFR of the 'Josho' cultivar tended to be higher than those of the other cultivars, particularly 'S-30' and 'Yusho' in later periods of the experiment. Data are means of 5–6 plants with 95% CIs.

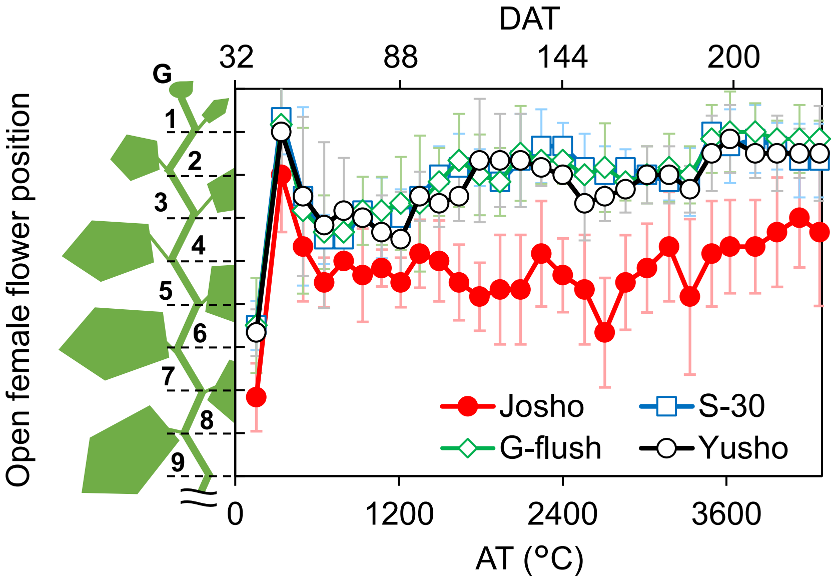

Figure S3. Position of an open female flower. Schematic diagram of a cucumber shoot, with nodes numbered from the growing point (G). The highest node bearing an open female flower indicates the flower position. AT, accumulated temperature. Data are means of six plants with 95% CIs.

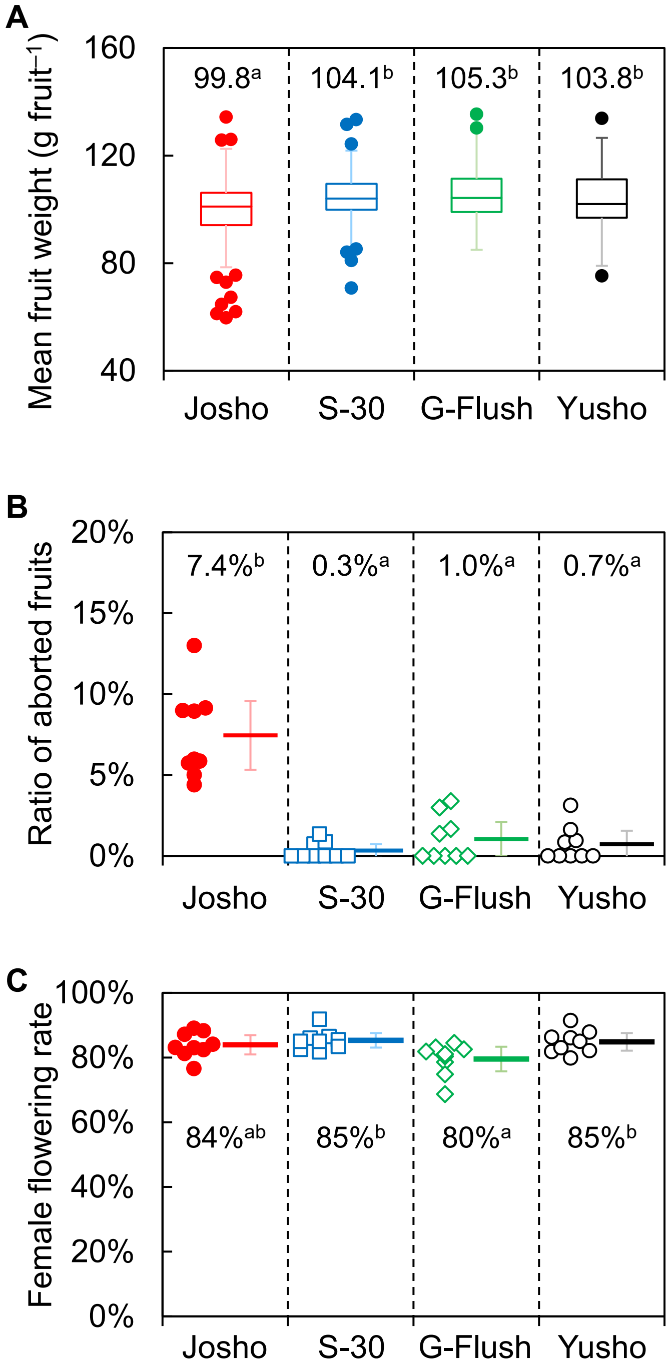

Figure S4. Harvested fruit weight, fruit abortion, and female flowering. (A) Box plot representing mean fruit weight in yield analysis. The mean weight was calculated for each harvest day (n = 183–185). Box represents interquartile ranges (IQRs) and whiskers indicate ranges within 1.5-fold IQR from the 1st and 3rd quartiles. Dots and values in the graph are outliers and means, respectively. (B) Ratio of aborted fruits. (C) Female flowering rate. Each plot represents an individual plant (n = 9 per cultivar). Horizontal bars and values in the graph represent means. Error bars indicate 95% CIs. Different letters indicate significant differences among cultivars ($P < 0.05$; Tukey's test).

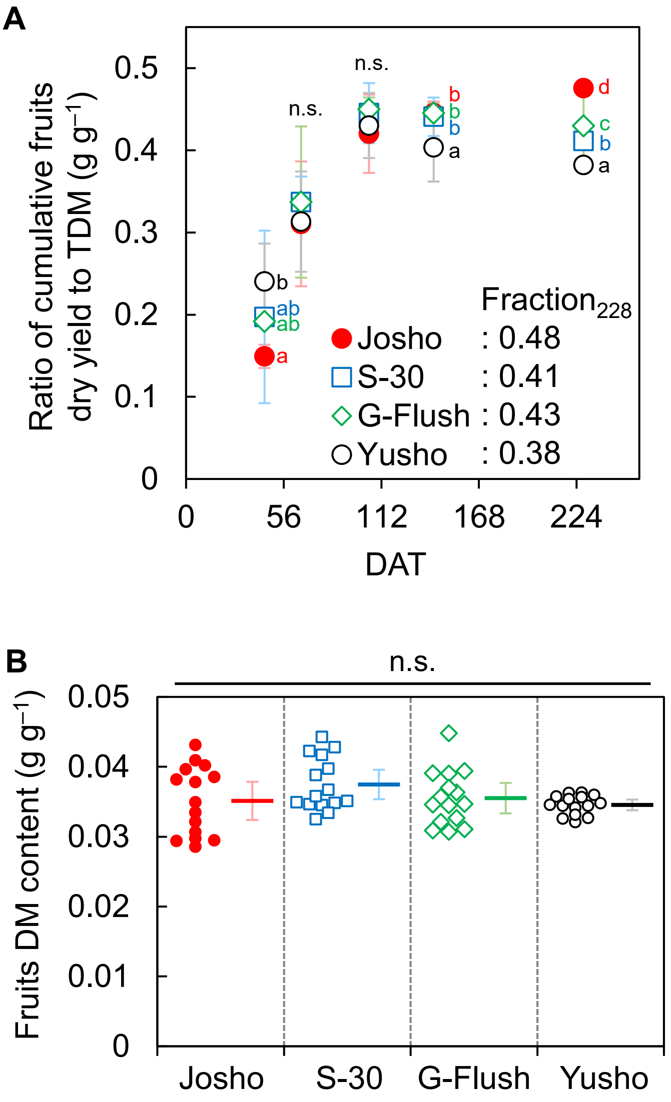

Figure S5. DM allocation to fruits and fruit quality. (A) Proportion of TDM allocated to fruits at each destructive sampling point. Different letters indicate significant differences among cultivars on the same date (P < 0.05; Tukey's test). Fraction$_{228}$ represents the mean value at the end of cultivation. (B) Fruit DM content at harvest. Each plot represents an individual fruit (n = 15 per cultivar). Horizontal bars and error bars indicate means and 95% CIs, respectively. n.s., not significantly different (P > 0.05; Tukey's test).

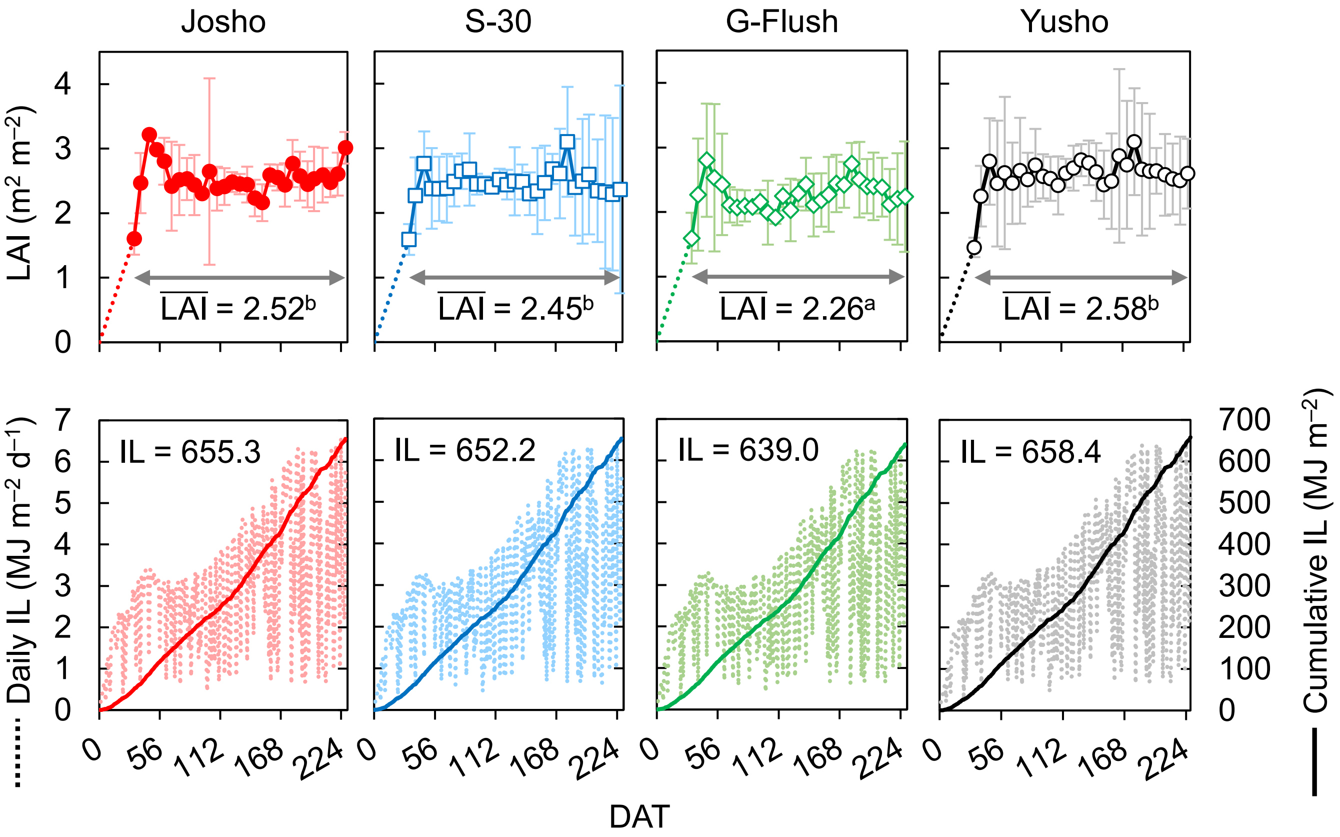

Figure S6. LAI and estimated light captured by the canopy. (A) Comparison of LAI values. Data are means of three plants with 95% CIs. Dotted lines indicate extrapolated values prior to the first measurement, assuming that LAI at transplanting was zero. The average LAI over the observation period is shown in the graph. (B) Estimated daily and cumulative light interception by the canopy. Final cumulative values at the end of cultivation are indicated.

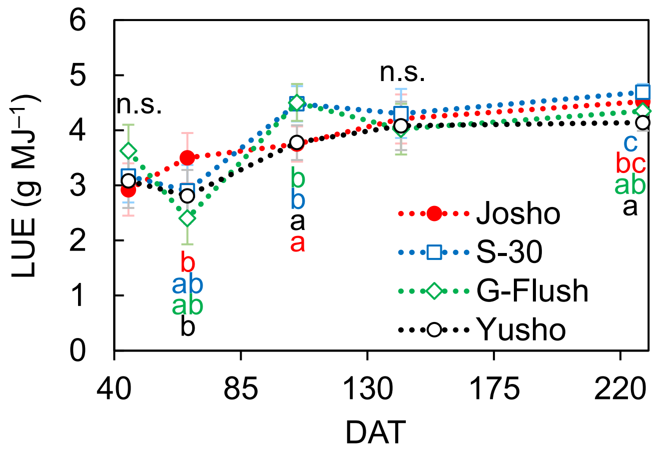

Figure S7. Changes in LUE. LUE was estimated for each term defined in Figure 4. Data are means and 95% CIs. Different letters indicate significant differences among cultivars within the same term ($P < 0.05$; Tukey's test). n.s., not significant.

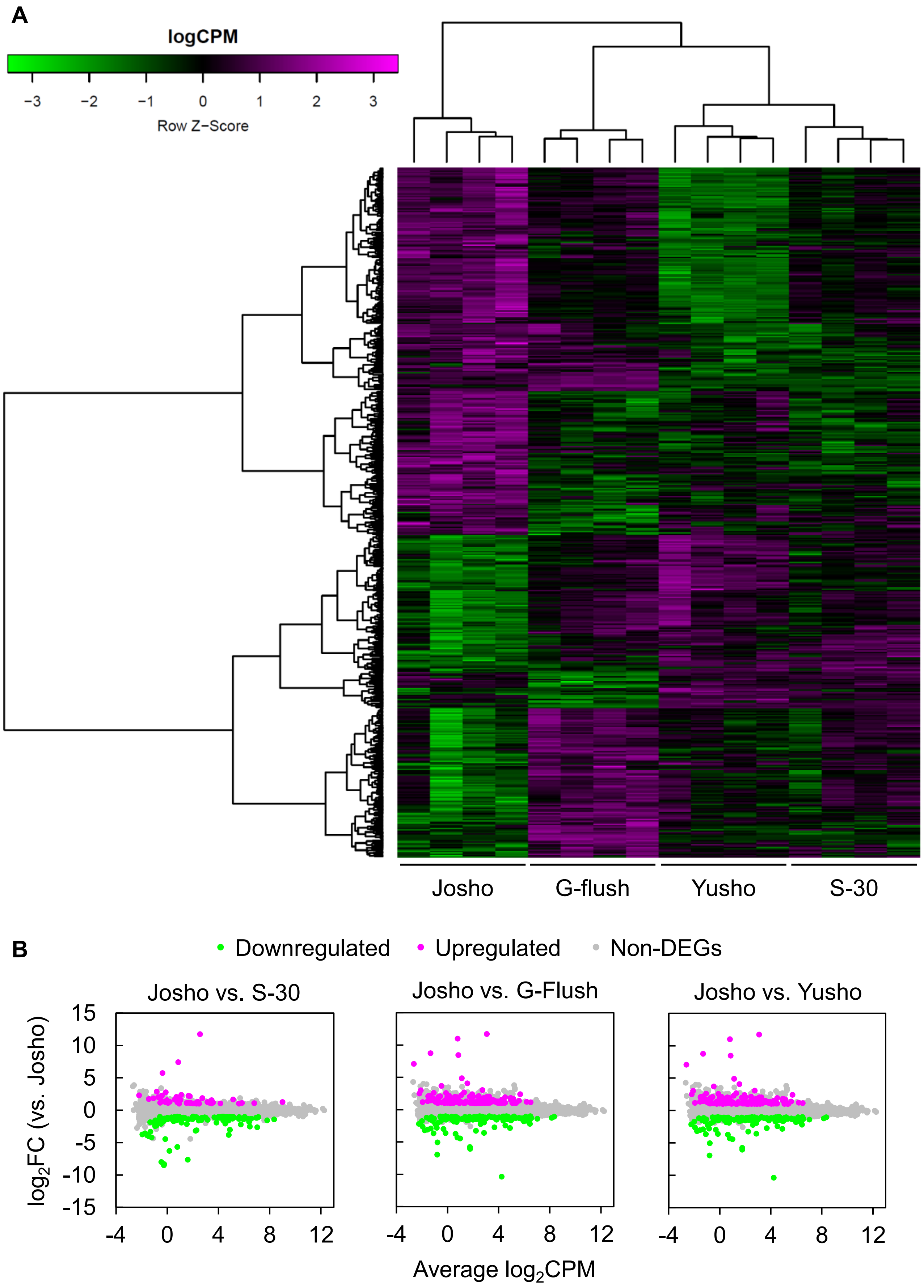

Figure S8. Differential gene expression analysis. (A) Hierarchical clustering of 513 DEGs using Kendall's tau distance and the Ward D2 clustering algorithm. (B) MA plots corresponding to Figure 7A.

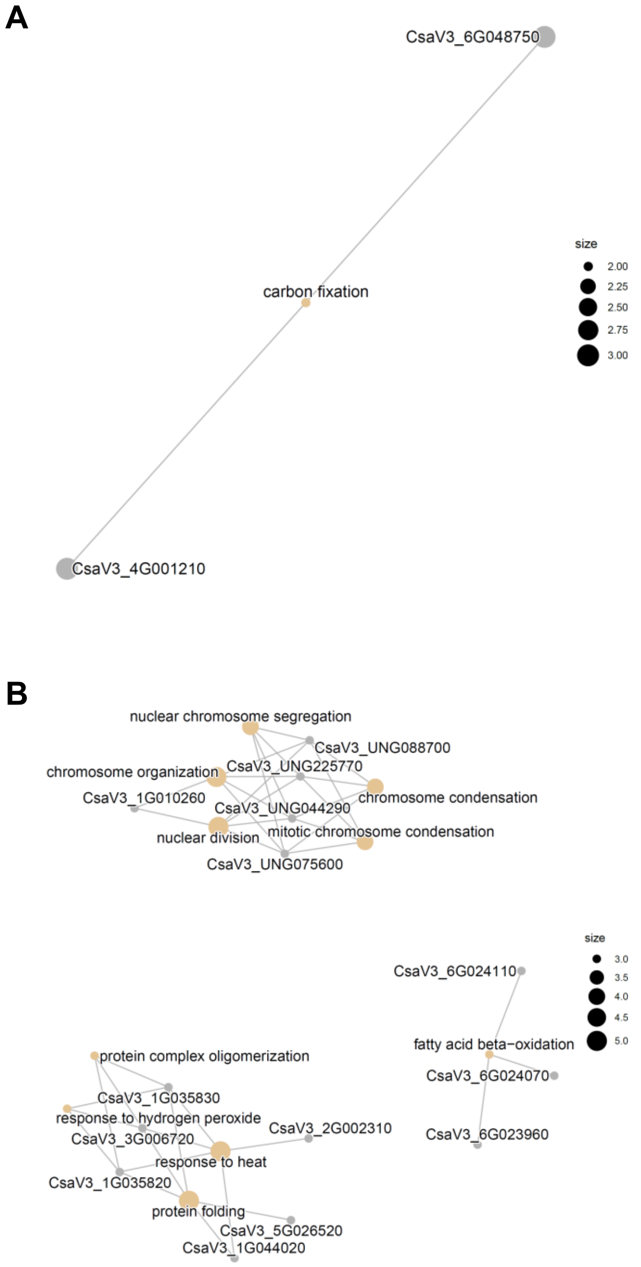

Figure S9. Common DEGs involved in the top 10 enriched GO terms. Downregulated (A) and upregulated (B) genes are shown as a network plot.
