# Supplementary material for: Comparative analyses of cucumber (Cucumis sativus L.) cultivars with varying node formation rate in greenhouse
Source: BMC Plant Biol. 2025 Nov 12;25:1547. doi: 10.1186/s12870-025-07587-3 (PMC12613712; doi:10.1186/s12870-025-07587-3)
Supplement: Supplementary file 2 — Supplementary Material 2. [file 12870_2025_7587_MOESM2_ESM.docx]

# Supplemental methods

## Additional short-term cultivation for RNA extraction and fruit DM content estimation

Commercially obtained seeds of the cucumber cultivars ‘Josho’, ‘S-30’, and ‘Yusho’ were sown in culture soil (TM-1; Takii Co., Kyoto, Japan), kept in the dark at 28°C for 1 day to promote germination, and then grown in a growth chamber (NAE Terrace; Mitsubishi Chemical Aqua Solutions, Tokyo, Japan) for 24 days. The chamber was set to a 14 h photoperiod, CO_2_ concentrations of 1000/400 ppm (day/night), and air temperatures of 28°/20°C (day/night). A commercially available nutrient solution (High-Tempo; Sumitomo Chemical, Tokyo, Japan) with an adjusted electrical conductivity of 1.4 mS cm^–1^ was supplied to the seedlings. On January 9, 2024, the seedlings were transplanted onto rock wool (Grodan, Roermond, Netherlands) in a greenhouse at NARO. Plants were grown with a single shoot at a density of 3.125 plants m^–2^ (raw and plant spacing: 1.6 m and 0.2 m, respectively) and irrigated with OAT-SA nutrient solution (OAT Agrio Co., Tokyo, Japan). The greenhouse environment was regulated using the Priva Maximizer system (Priva, De Lier, Netherlands). Fruits harvested between 66 and 70 DAT were used to estimate fruit DM content. Shoot growth was monitored from 21 to 98 DAT, during which the daily average temperature, mean daytime CO_2_ concentration, and mean outside solar radiation were 18.6–23.9°C, 453 ppm, and 13.7 MJ m^–2^, respectively.

**Table S1.** Accession numbers of genes functioning in the shoot apical meristem.

| **Category** | **Gene name** | **Accession number** | | **Version** | **Reference** |
| --- | --- | --- | --- | --- | --- |
|  |  | **Current** | **Old** |  |  |
| PEBPs | *CsFT* | *CsaV3_1G044210* | *Csa1G651710* | V2 | [1] |
|  | *CsTFL1* | *CsaV3_6G040640* | *Csa6G452100* | V2 | [1] |
|  | *CsTFL1d* | *CsaV3_6G014990* | *Csa6G152360* | V2 | [2, 3] |
| FT/TFL1 complex subunits | *CsNOT2a* | *CsaV3_6G022060* | *Csa6G302150* | V2 | [1] |
|  | *CsFD* | *CsaV3_3G000600* | *Csa3G002610* | V2 | [1] |
|  | *CsFDP* | *CsaV3_6G004560* | *Csa6G051480* | V2 | [1] |
|  | *CsGF14-3* | *CsaV3_2G029820* | *Csa2G369070* | V2 | [1] |
|  | *CsGF14-5* | *CsaV3_4G008800* | *Csa4G094520* | V2 | [1] |
| SAM maintenance &  FM differentiation | *CsLFY* | *CsaV3_1G000050* | *Csa1G000050* | V2 | [4] |
|  | *CsWUS* | *CsaV3_6G047050* | *Csa6G505860* | V2 | [4] |
|  | *CsUFO* | *CsaV3_1G010110* | *Csa1G056950* | V2 | [4] |
|  | *CsHAN1* | *CsaV3_4G005580* | *Csa016191* | V1 | [5] |
|  | *CsHAN2* | *CsaV3_6G046280* | *Csa012029* | V1 | [5] |
|  | *CsSTM* | *CsaV3_6G048730* | *Csa000554* | V1 | [5] |
|  | *CsBP* | *CsaV3_4G035390* | *Csa009344* | V1 | [5] |

Accession numbers were identified by BLAST searches of corresponding amino acid sequences available in the old reference database (http://cucurbitgenomics.org/). FM, floral meristem.

**References**

1. Wen *et al.* Development, 2019; **146**: dev180166
2. Njogu *et al*. Theor. Appl. Genet., 2020; **133**: 3323–3332
3. Wen *et al*. Plant Cell Environ., 2021; **44**: 2580–2592
4. Zhao *et al*. New Phytol., 2017; **218**: 344–356
5. Ding *et al*. J. Exp. Bot., 2015; **66**: 7075–7087
